# Supplementary material for: ProteinShader: illustrative rendering of macromolecules
Source: BMC Struct Biol. 2009 Mar 30;9:19. doi: 10.1186/1472-6807-9-19 (PMC2672931; doi:10.1186/1472-6807-9-19)
Supplement: Additional file 1 — ProteinShader program without source code. This compressed file contains the complete ProteinShader program including associated libraries, but no source code. A README.txt file gives an overview of the ProteinShader distribution, and the index.html file in the help subdirectory has directions on getting started with the program as well as a set of tutorials. [file 1472-6807-9-19-S1.zip › ProteinShader-beta-0_9_4-binary/help/api/org/proteinshader/structure/class-use/Structure.html]

Uses of Class org.proteinshader.structure.Structure (ProteinShader API)


|  |  |  |
| --- | --- | --- |
| **Overview** \| **Package** \| **Class** \| **Use** \| **Tree** \| **Deprecated** \| **Index** \| **Help** | |  |
| PREV   NEXT | **FRAMES**    **NO FRAMES**     **All Classes** |  |


---


## **Uses of Class org.proteinshader.structure.Structure**

| Packages that use Structure | |
| --- | --- |
| **org.proteinshader.gui** | Holds all of the Swing GUI components and their associated listeners, including class Renderer, which is registered as a listener for the GLCanvas object that is used a drawing surface. |
| **org.proteinshader.gui.components.controlpanel** | Holds the JPanels used in the right-side retractable control panel of the GUI. |
| **org.proteinshader.structure.io** | Holds the classes needed for reading Protein Data Bank structure files. |
| **org.proteinshader.structure.io.demos** | Holds demo classes that were used for early testing of the io classes. |
| **org.proteinshader.structure.visitor** | Holds Visitor classes that know how to traverse the hierarchy of objects held by class Structure. |
| **org.proteinshader.structure.visitor.demos** | Holds demo classes that were used for early testing of the Visitor classes. |

Uses of Structure in org.proteinshader.gui

| Methods in org.proteinshader.gui that return Structure | |
| --- | --- |
| `Structure` | `MediatorImpl.getStructure()`             Returns the current Structure to display. |
| `Structure` | `Mediator.getStructure()`             Returns the current Structure to display. |

Uses of Structure in org.proteinshader.gui.components.controlpanel

| Methods in org.proteinshader.gui.components.controlpanel with parameters of type Structure | |
| --- | --- |
| `void` | `ControlPanel.addNewStructure(Structure structure)`             The Structure is used to set the Model menu of the SelectorPanel, which will automatically set the other menus and lists of the SelectorPanel. |
| `void` | `SelectorPanel.setModelMenu(Structure structure)`             Adds the Structure's Models to the Model menu. |

Uses of Structure in org.proteinshader.structure.io

| Methods in org.proteinshader.structure.io that return Structure | |
| --- | --- |
| `Structure` | `StructureReader.readStructure(File file)`             Reads a protein structure from a file and returns a Structure object. |
| `Structure` | `PDBStructureReader.readStructure(File file)`             Reads a protein structure from a ".pdb" file and returns a Structure object. |
| `Structure` | `StructureReader.readStructure(String filename)`             Reads a protein structure from a file and returns a Structure object. |
| `Structure` | `PDBStructureReader.readStructure(String filename)`             Reads a protein structure from a ".pdb" file and returns a Structure object. |

Constructors in org.proteinshader.structure.io with parameters of type Structure

`PDBLineParser(Structure structure)`
  
          Constructs a PDBLineParser for the Structure given as an argument.

Uses of Structure in org.proteinshader.structure.io.demos

| Methods in org.proteinshader.structure.io.demos with parameters of type Structure | |
| --- | --- |
| `void` | `PDBStructureReaderDemo.writeStructure(Structure structure, String filename)`             Writes a Structure to a file by using a SFWriterVisitor. |
| `void` | `SegmentDemo.writeStructure(Structure structure, String filename)`             Writes a Structure to a file by using a SFWriterVisitor. |

Uses of Structure in org.proteinshader.structure.visitor

| Methods in org.proteinshader.structure.visitor with parameters of type Structure | |
| --- | --- |
| `void` | `Visitor.visit(Structure structure)`             Traverses all Models owned by the Structure. |
| `void` | `SFWriterVisitor.visit(Structure structure)`             Writes a Structure's data to a simple text file. |
| `void` | `BondGeneratorVisitor.visit(Structure structure)`             Generates all standard Bonds (AminoAcids and Waters) for the Structure. |

Uses of Structure in org.proteinshader.structure.visitor.demos

| Methods in org.proteinshader.structure.visitor.demos with parameters of type Structure | |
| --- | --- |
| `void` | `ModifierVisitorDemo.modify(Structure structure)`             The Helices of the Structure are modified by changing their shape to CYLINDER and setting all of their Atoms and Bonds to INVISIBLE. |
| `void` | `VisitorDemo.testVisitor(Structure structure)`             Creates a Visitor and tests it by activating debug mode so that it will print the ID of each object in the Structure. |
| `void` | `ModifierVisitorDemo.writeStructure(Structure structure, String filename)`             Writes a Structure to a file by using a SFWriterVisitor. |

---


|  |  |  |
| --- | --- | --- |
| **Overview** \| **Package** \| **Class** \| **Use** \| **Tree** \| **Deprecated** \| **Index** \| **Help** | |  |
| PREV   NEXT | **FRAMES**    **NO FRAMES**     **All Classes** |  |


---

# *Copyright © 2007-2008*
